# Heterotachy in Mammalian Promoter Evolution

**DOI:** 10.1371/journal.pgen.0020030

**Published:** 2006-04-28

**Authors:** Martin S Taylor, Chikatoshi Kai, Jun Kawai, Piero Carninci, Yoshihide Hayashizaki, Colin A. M Semple

**Affiliations:** 1 Wellcome Trust Centre for Human Genetics, University of Oxford, Oxford, United Kingdom; 2 Genome Exploration Research Group, RIKEN Genomic Sciences Center, RIKEN Yokohama Institute, Yokohama, Japan; 3 Genome Science Laboratory, Discovery Research Institute, RIKEN Wako Institute, Wako, Japan; 4 Medical Research Council Human Genetics Unit, Western General Hospital, Edinburgh, United Kingdom; The Jackson Laboratory, US; MRC-Harwell, UK; NHGRI-NIH, US; Lawrence Livermore National Laboratory, US; The Jackson Laboratory, US

## Abstract

We have surveyed the evolutionary trends of mammalian promoters and upstream sequences, utilising large sets of experimentally supported transcription start sites (TSSs). With 30,969 well-defined TSSs from mouse and 26,341 from human, there are sufficient numbers to draw statistically meaningful conclusions and to consider differences between promoter types. Unlike previous smaller studies, we have considered the effects of insertions, deletions, and transposable elements as well as nucleotide substitutions. The rate of promoter evolution relative to that of control sequences has not been consistent between lineages nor within lineages over time. The most pronounced manifestation of this heterotachy is the increased rate of evolution in primate promoters. This increase is seen across different classes of mutation, including substitutions and micro-indel events. We investigated the relationship between promoter and coding sequence selective constraint and suggest that they are generally uncorrelated. This analysis also identified a small number of mouse promoters associated with the immune response that are under positive selection in rodents. We demonstrate significant differences in divergence between functional promoter categories and identify a category of promoters, not associated with conventional protein-coding genes, that has the highest rates of divergence across mammals. We find that evolutionary rates vary both on a fine scale within mammalian promoters and also between different functional classes of promoters. The discovery of heterotachy in promoter evolution, in particular the accelerated evolution of primate promoters, has important implications for our understanding of human evolution and for strategies to detect primate-specific regulatory elements.

## Synopsis

Promoters are crucial to the regulation of gene expression. They are of considerable interest to molecular biologists from a functional perspective and to a much wider audience, as sequence changes within promoters are likely to be a substantial contributor to disease predisposition and the divergence of species. In mammals, promoters have been extensively studied in a case-by-case manner, but the more general mechanisms of promoter evolution are little understood. The authors have undertaken an extensive study of evolutionary trends across experimentally defined promoters. They have discovered that the relative rate of promoter evolution varies between lineages and is substantially accelerated in primates. The authors conclude that the predominant cause is variation in the mutation rate specifically within promoter regions. This finding has important implications for comparative genomics, in particular the identification of functional sites within promoters. The large datasets in this study have also allowed the pattern of evolution to be considered between different types of promoter, giving new insight into their distinct biology.

## Introduction

Although promoter architecture is complex in multicellular eukaryotes two key features seem to be universally shared: (i) a basal/core promoter region perhaps 100 bp upstream of the transcription start site (TSS) [1] and (ii) various widespread transcription factor binding sites (TFBSs) conferring specificity of transcription, generically referred to as enhancers. *Cis*-regulatory elements as far as ~1 Mb from the core promoter have been found [2], though the discovery and validation of regions so distant from the genes they influence presents substantial challenges. However, it seems that a proximal promoter region (~500 bp from the TSS) usually possesses all activity necessary to direct expression. It has been shown that 91% of the putative promoters derived from the 550 bp of genomic sequence immediately upstream of a collection of full-length cDNA clones have promoter activity when assayed using luciferase-based transfection in four human cultured cell types [3]. Furthermore, in similarly selected putative promoters, around a third of identified single nucleotide polymorphism variants resulted in altered expression [4].

Investigations of the regions immediately upstream of known TSS positions have successfully identified functional TFBSs, using combinations of motifs representing the specificity of a TFBS and “phylogenetic footprinting” [5,6]. In comparisons of mouse, rat, and human orthologous sequences it has been shown that phylogenetic footprinting can allow a 44-fold reduction in the number of false positive matches to TFBS motifs [7]. The basis for the success of phylogenetic footprinting is well established; functional regulatory regions are more highly conserved than neutrally evolving sequences, presumably a result of purifying selection [8]. Thus, the use of comparative genomics to estimate broad evolutionary constraints, often in terms of the best-conserved regions, is widespread. Indeed, applying this practice to modest numbers of promoters is now commonplace. However, we lack a more general account of the molecular evolutionary dynamics and mechanisms governing promoter divergence.

Although there have been many studies of promoters within particular pairs of orthologous genes, to date there have been few larger scale studies of mammalian promoter evolution, because of the shortage of experimentally supported TSS positions. Most of these studies have used sequences upstream of start codons as a surrogate for defined TSSs and may therefore have included transcribed 5′ untranslated regions and intronic sequences, in some cases entirely missing the functional promoter. Jareborg et al. [9] found evidence for selective constraint in the promoters of 77 genes, in the form of conserved regions (>60% identity over 100 bp between human and mouse) including 36% of the promoter sequence they examined. Another study found that 10% of nucleotides were selectively constrained even in alignments of very long intergenic regions from 100 mouse and human genomic regions [10]. Keightley and Gaffney [11] examined evolutionary constraints at 300 orthologous loci in mouse and rat and showed that on average selective constraints on coding sequences are around an order of magnitude stronger than on upstream sequences. More recently, a study of divergence at 1,000 primate loci and 300 rodent loci made the surprising observation that promoter regions lack detectable selective constraint in the primate lineage [12]. Indeed, selective constraint appears to be weak across all conserved nongenic regions of the primate genome [13,14].

In spite of the modest data available, it is clear that variation in transcriptional regulation constitutes a significant part of the raw material for phenotypic evolution [15,16]. This variation is generated by various mutations that distinguish its evolution from that of coding sequence. As with coding sequence, there are regions of promoters that diverge through single nucleotide substitutions, but a variety of other mutations are also relevant. As with all noncoding sequences, small insertion or deletion (indel) events can also play important roles. Expansions or contractions in microsatellite repeat arrays can alter the number of and spacing between functional binding sites [15]. Larger scale rearrangements such as transposition [17] and duplication [16] can also assemble novel regulatory sequences. Indeed, almost 25% of human promoter regions reportedly contain transposable element insertions, and it is known that some experimentally characterised *cis*-regulatory elements have been derived from such sequences [18]. Any comprehensive study of promoter evolution must therefore examine substitution rates in parallel with these less gradual mutations.

Comparative analyses of mammalian promoters have typically used translational start sites as surrogates for TSSs. However, there are now unparalleled opportunities to study mammalian promoter evolution afforded by the availability of reliable whole genome alignments [19] and high-quality measures of TSS positions [20]. Here we undertake to our knowledge the largest ever survey of evolutionary divergence in mammalian promoters using large sets of mouse and human promoters based upon experimentally validated TSSs. We demonstrate the relative selective constraint that has operated in different lineages and also provide substitution rate measures for core and more distal promoter regions. We also discuss the differences in these parameters for various broad categories of promoters such as those with and without TATA boxes and those with and without CpG islands. In addition, we investigate the roles of indel rates and repeat insertions in promoter evolution.

## Results/Discussion

### The Mutational Spectrum and Divergence of Mammalian Promoters

Based on 30,969 mouse and 26,341 human experimentally validated TSSs [20] we surveyed the broad characteristics of evolutionary divergence in mammalian promoters. These large mouse and human TSS datasets allowed us to examine the extent of positional conservation in TSSs across mammals. We assessed the overlap between mouse and human TSSs within mouse–human whole genome alignment data [19]. For 71% of mouse TSSs that aligned with human, we found a corresponding TSS in the orthologous sequence. This indicates that TSSs defined in mouse are likely to correspond to functional TSSs in humans and the other mammalian genomes in this study. However, this is not an explicit assumption in the following analyses; rather, we are measuring the past constraint of sequences that currently drive transcription in a genome.

To provide a model of near-neutral sequence evolution for comparison to promoter sequences, we also analysed 14,460 ancient repeat sequences (ARs). A recent estimate for substitutions per site *(K)* between mouse and rat genomes at a range of selectively neutral sites was 0.174, with the equivalent rates between mouse and human genomes being 0.493 [7] and between human and chimpanzee being 0.0122 [21]. Unfortunately, these estimates lacked conventional measures of variation and were made for a variety of putatively selectively neutral sites: ARs, 4-fold degenerate sites in codons, and rodent-specific sites [7]. Additional neutral substitution rate estimates for mouse versus human and mouse versus rat were reported as 0.552 and 0.196, respectively [22], but again these estimates lack accepted estimates of variation and are based on methodology that differs from our own (in the alignment algorithm used, the sites studied, and the model of evolution assumed). It is therefore not possible to make a statistically rigorous comparison between our results and these previous estimates. However, our rate estimates for the same species comparisons in ARs were 0.175 (95% confidence interval [±] 0.0003) and 0.526 ± 0.001, respectively, and we derived 0.0127 ± 0.0001 for human versus chimpanzee, which suggests that our substitution rate estimates are broadly in line with other large-scale studies. Our rate estimates are also consistent with the accepted phylogeny relating the organisms under study [23,24]: human, chimpanzee, rhesus macaque, mouse, rat, and dog. For example, for all mouse-based alignments the divergence of mouse versus rat was less than that of mouse versus human, which was less than that of mouse versus dog.

**Figure 1:**
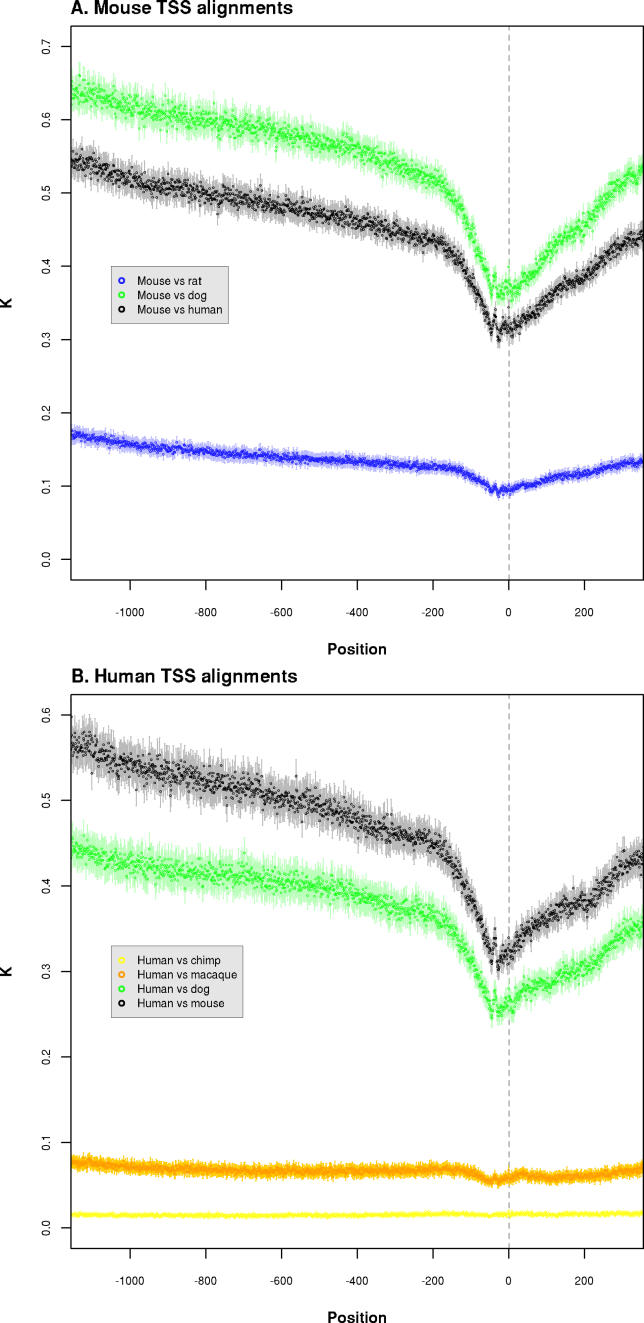
High-Resolution Pairwise Substitution Rate Estimates *(K)* across Promoter Region Alignments The *x-*axis denotes nucleotide position relative to the TSS reference position at +1 (grey vertical line). Error bars (lighter shading) show 95% confidence intervals for each data point. (A) Rates calculated from mouse-based alignments. (B) Rates calculated from human-based alignments.

**Figure 2:**
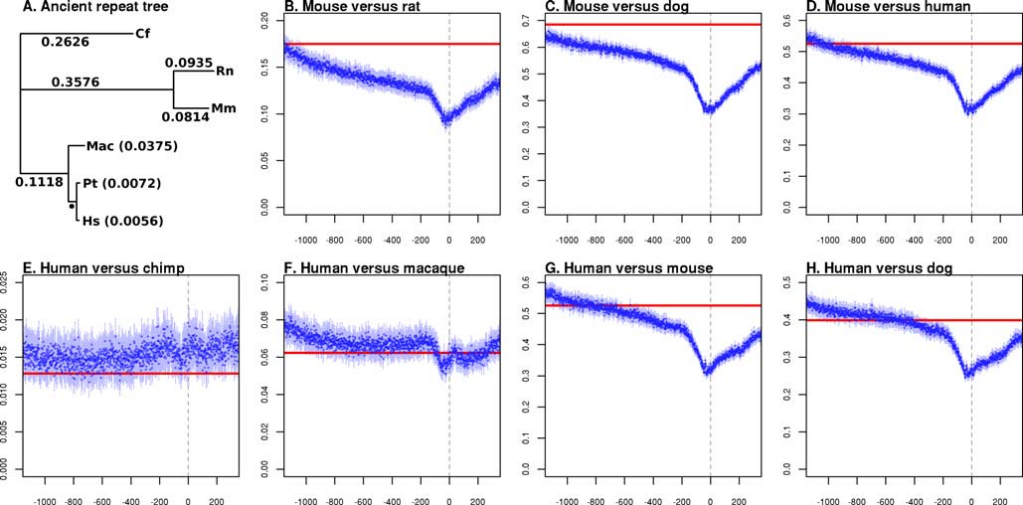
Relative Selective Constraint across Mammalian Promoters (A) Nucleotide substitution rates (*K,* substitutions per aligned nucleotide) calculated from AR alignments. Rates for each branch are shown along the branch where possible, otherwise in parentheses after the species abbreviation. A single black spot indicates the branch length 0.0192, which could not be accommodated on the graph. (B–H) Pairwise substitution rate estimates (with 95% confidence intervals indicated) showing both the substitution rate (*K*, *y-*axis) calculated from ARs (red) and at each nucleotide position across the promoter region (position shown on the *x-*axis). In every case, the 95% confidence interval for ARs is contained within the plotted line. The TSS position at +1 is indicated by a grey vertical line. (B–D) Mouse-based alignments of TSSs defined in mouse. (E–H) Human-based alignments of TSSs defined in human. Cf, *Canis familiaris;* Hs, *Homo sapiens;* Mac, *Macaca mullata;* Mm, *Mus musculus;* Pt, *Pan troglodytes;* Rn, Rattus norvegicus.

We have calculated substitution rates across promoter regions at single nucleotide resolution, from nucleotide positions −1,100 to +300 relative to the TSS at the +1 position (Figure 1). In comparisons between nonprimate mammals, *K* is minimal in the ~50 nucleotides (nt) upstream of the TSS (Figure 1A), then increases rapidly within 200 nt of the TSS position. *K* continues to rise steadily further upstream until reaching a substitution rate that does not differ significantly from that derived from ARs (Figure 2A and 2B). With the exception of human versus chimpanzee rate estimates, the overall pattern of *K* across promoter regions is consistent, indicating that the first 200 nt upstream of the TSS is a good approximation to the classically defined core promoter region [1]. In regions further upstream, the frequency of selectively constrained sites when averaged across all promoters diminishes as a linear function of the distance to the TSS (Figures 1 and 2). However, it is striking that in almost every comparison involving primates, the substitution rate of the TSS upstream sequence (−200 to −1,100) is either constantly above the neutral rate calculated from ARs (human–chimpanzee and human–macaque) or rises above it (Figure 2; Tables 1 and 2). This is a feature not only of human defined TSSs but also of mouse TSSs in pairwise comparison with human (Figure 2D). The human–chimpanzee comparisons differ in that the substitution rate is highest just 200 nt upstream of the TSS, and is consistently measured to be above the rate estimated from ARs (Figure 2E). We return to these observations for detailed analysis in the next section, but first we consider the spectrum of indel mutations, and the contribution of repetitive elements to mammalian promoter evolution.

**Table 1:**
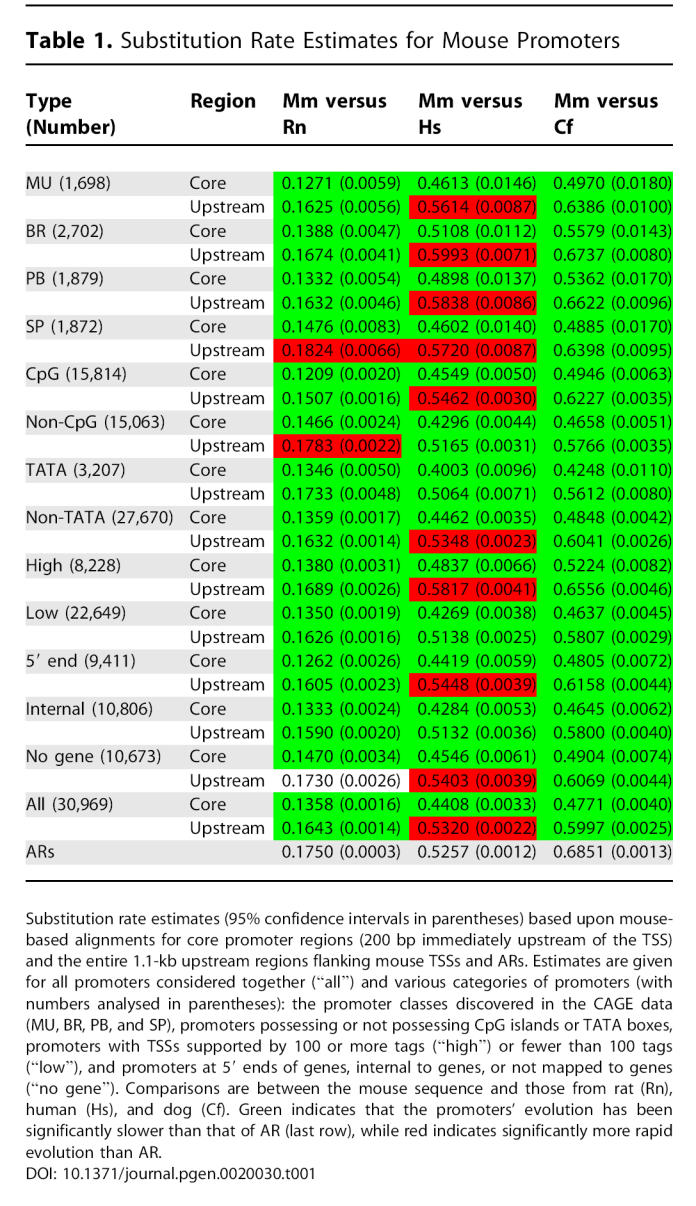
Substitution Rate Estimates for Mouse Promoters

**Table 2:**
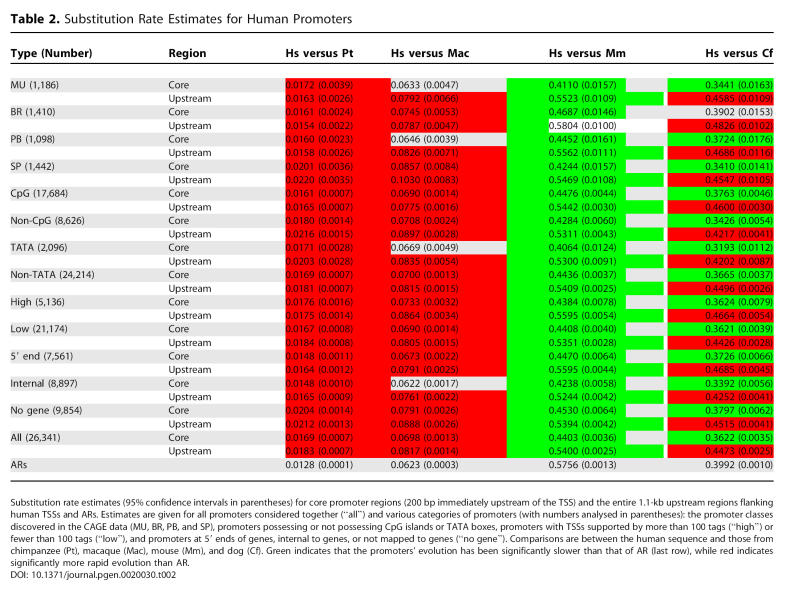
Substitution Rate Estimates for Human Promoters

Micro-indels (<11 nt) are common mutational events in genomes [7], but the mechanisms giving rise to them and their consequences for evolution can differ markedly from those of nucleotide substitutions [25,26]. It is therefore likely that these events are an important, though until now little studied, component of the mutational spectrum in promoters. We have taken a conservative approach to identifying indels and assigning them to a particular lineage (see Materials and Methods). This certainly underestimates the absolute rate of indel mutation and fixation, but does allow meaningful measurement of relative rates between regions of a genome. Importantly, we have only considered indels that can be resolved to the mouse or human terminal branch (lineage from the mouse–rat common ancestor to mouse and from the human–chimpanzee ancestor to human) to avoid uncertainties introduced by the lower quality draft status genomes we have included.

**Figure 3:**
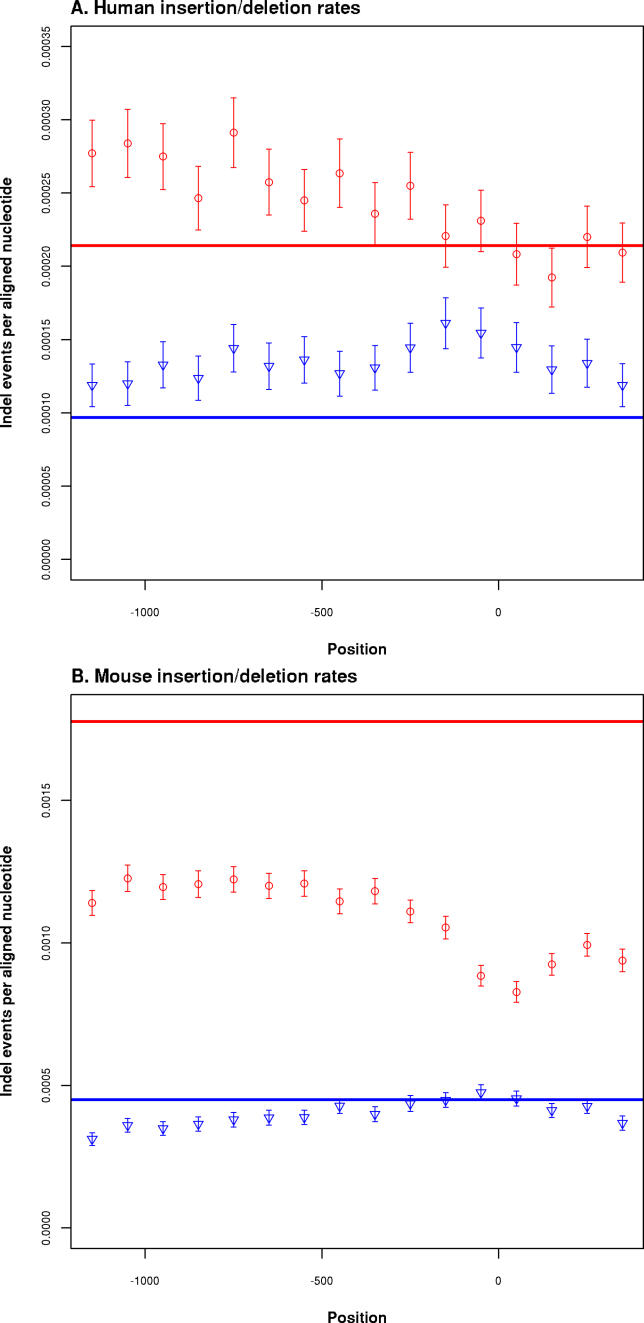
Micro-Insertion and -Deletion Rates Promoter rates calculated as insertion (blue) and deletion (red) events per nucleotide in 100-bp consecutive windows (*x-*axis). Error bars show 95% confidence intervals; solid horizontal lines show rates calculated from AR alignments. Vertical grey line indicates the +1 TSS position. (A) Human rates based on alignments between human, chimpanzee, and macaque; rates shown are derived only from the human terminal branch (see Materials and Methods). (B) Mouse terminal branch rates based on comparisons between mouse, rat, and dog.

As with substitution rates, we find the lowest rate of deletions in the regions immediately adjacent to the TSS, indicating the action of purifying selection. In the mouse terminal branch, the deletion rates across the entire 1.3-kb promoter-encompassing regions analysed are significantly below that for the AR rate (Figure 3A). This is a substantially more pronounced effect than that observed for substitution rates, suggesting that deletions are on average more deleterious than substitutions in promoter regions. In the human terminal branch, we see a similar pattern, with a clear reduction in deletion rate in the core promoter and first exon regions relative to more distal promoter and downstream sites (Figure 3B). Remarkably, as with primate substitution rates in human promoters, the deletion rate is either not significantly different from that of ARs, or in further upstream regions significantly exceeds that of ARs.

In contrast to the pattern of substitutions and deletions, the rate of micro-insertion shows a general upward trend as the core promoter region is approached from either the upstream or downstream side (Figure 3). There is no evidence for the expected selection against insertions in the core promoter region or immediately downstream of the TSS. This pattern could be construed to indicate a lack of selection against insertions, or even positive selection for insertions in promoter regions. We consider this unlikely, as it would require sustained positive selection in a large fraction of promoters specifically for insertions and not other mutational events. Rather, we suspect that the elevated insertion rate in the core promoter represents the previously reported positive correlation between CpG dinucleotide frequency and insertion rate [26]. Supporting this, we note that masking annotated CpG islands diminishes, although does not fully remove, the trend (data not shown).

As with both substitution and deletion rates, promoter region insertion rates in the human lineage significantly exceed those calculated from ARs. In general, the mouse lineage insertion rates are below those of ARs but do rise such that they are not significantly different within ~100 nt of the TSS. It is also notable that in both promoter regions and ARs, the frequency of deletions is always significantly greater than the frequency of insertions. This is consistent with previous findings for the rodent lineage [7] and confirms that an excess of small deletions over insertions is also a property of primate genome evolution.

The transposition of repetitive elements is thought to be a major force in genome evolution, and sequences derived from such elements have been co-opted to drive the transcription of cellular genes [18]. Table 3 summarises the density of the main repeat classes for mouse and human promoters compared with the density expected given the genome-wide frequencies for each class. A significant excess of the RNA class was found in both mouse and human promoter regions. This class contains matches to small structural RNA genes that are responsible for a large number of processed pseudogenes in mammals. The greater than expected density of the RNA class in both human and mouse is likely to be a result of the tendency for these pseudogenes to be concentrated in gene-rich, transcriptionally active regions of the mammalian genome [27]. No other repeat classes are significantly over-represented in either mouse or human core promoter regions, and, in fact, for most repeat classes the number of repeats observed was significantly below that expected (Table 3). The same pattern is seen in human core promoters for SINEs in spite of SINEs being the most common interspersed repeat class in the human genome and showing preferential retention in GC-rich sequence [28]. This suggests that both human and mouse promoters are refractory to large mutations such as interspersed repeat insertion, with purifying selection acting to remove integrations in both lineages.

**Table 3:**
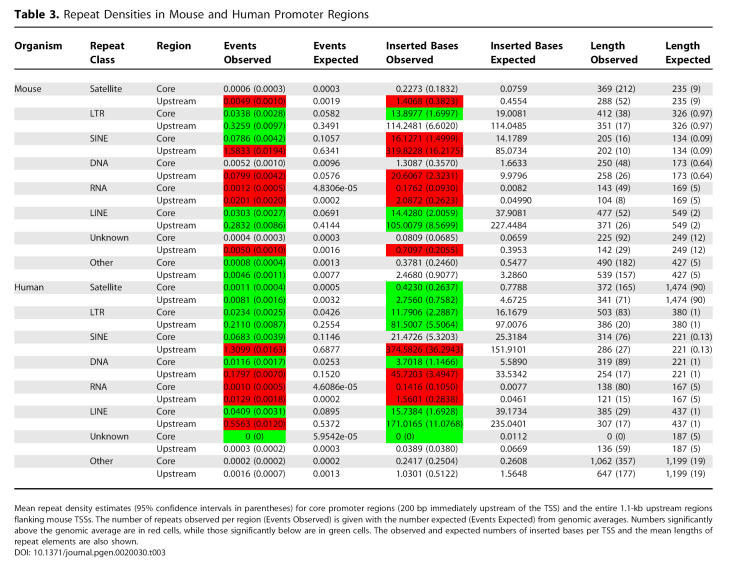
Repeat Densities in Mouse and Human Promoter Regions

### Accelerated Evolution of Primate Promoters

We have consistently found that substitution, insertion, and deletion rates in human promoters exceed those measured in ARs, which are assumed to be evolving in a nearly neutral manner (Figures 2 and 3). This apparent acceleration of primate promoter evolution is evident across all subcategories of promoter we identified (Tables 1 and 2), but is contrary to prior expectation. Promoters are functional elements that on average would be expected to be subject to purifying selection and so to evolve at a rate substantially slower than the neutral rate. The acceleration is also the opposite of what we find when investigating the mutational spectra of mouse promoters, in particular through comparison with nonprimate mammals (Figures 2 and 3). There are a number of possibilities that could explain these observations; they broadly fall into three categories: (i) the acceleration is an artefact due to the dominant effect of sequencing error in draft genome sequences; (ii) there is a higher background mutation rate in promoter regions than in ARs (this effect must be more pronounced in primates than rodents to explain our observations); or (iii) there is a general lack of constraint in primate promoters combined with positive selection at a subset of promoters.

The contiguity and nucleotide error rate of the six genomes used in this study are not all equal. The human, mouse, and rat genomes [7,29,30] are all extremely high quality assemblies in which each nucleotide has been sequenced many times. In contrast, the dog, macaque, and chimpanzee genomes [31,32] are currently much lower quality draft assemblies. The per-nucleotide error rate for the chimpanzee genome assembly used here is estimated to be 0.0001 for 98% of the sequence [32], whereas the divergence between human and chimpanzee we measure from AR is *K* = 0.013 (Figure 2A; Table 2). As sequence errors are approximately two orders of magnitude less frequent than real differences between these species, this suggests that in general the effect of sequence errors on substitution rate estimates is negligible, even between these closely related species. However, it is conceivable that the 2% of sequence most prone to error could be concentrated into promoter regions. We control for this possibility by considering only the human terminal branch when estimating substitution rates, that is, only calling a substitution if the sequence of, for example, both macaque and chimpanzee agree and human differs. Using human terminal branch estimates we still find upstream sequences significantly exceed the AR terminal branch rate (upstream: 0.0062 ± 0.00045; core: 0.0064 ± 0.001; AR: 0.0056 ± 0.00008). As previously noted, the same is also true for insertions and deletions (Figure 3B). Finally, comparisons between distantly related species such as human–dog, in which *K* and the estimated nucleotide error rate [31] are separated by more than three orders of magnitude (error rate < 0.0001 versus *K* = 0.3846), still show a substitution rate exceeding that of ARs for the more distal upstream promoter regions. We conclude from these lines of evidence that genome quality and sequence errors cannot explain the higher rate of substitutions (and indels) in human promoters than in ARs.

The elevated substitution rates in humans could be explained if there is a higher mutation rate in promoters than in ARs. This effect would have to be substantially greater in the human than the mouse lineage to explain the apparent confinement of this effect to comparisons involving human sequence. As the CpG dinucleotide is prone to a high rate of point mutation, and clusters of CpG are associated with a class of promoter (Tables 1 and 2), one could hypothesise that the apparent acceleration of primate promoter evolution is due to a high level of CpG-specific mutations. This idea is bolstered by the observation that the frequency, size, and CpG enrichment of CpG islands are greater in primate than rodent genomes [30], suggesting this effect may be more pronounced in primates than rodents.

To test this hypothesis directly, we considered the rate of transversion substitutions separately from that of transitions. Because the mutational mechanism that causes elevated substitution at CpG results specifically in transition mutations, the transversion rate should be unaffected by direct CpG effects. We found that transversion rates in primate promoter regions are significantly higher than in ARs (Figure S1), demonstrating clearly that the high relative *K* in primate promoters is not a consequence of direct CpG effects. Furthermore, the ratio of transition to transversion is tipped more in the favour of transversions in promoters than in ARs (upstream: 0.40; AR: 0.38), contrary to expectation for a dominant CpG influence. We also applied an independent method [12] to mask any sites that are likely to have mutated from CpG dinucleotides. Again, even after this masking we found that the substitution rates in both core promoters and upstream regions significantly exceed equivalent rates calculated from ARs (Table S1). Each of these lines of evidence is consistent with the observation that human promoters lacking CpG islands also show a higher than neutral substitution rate (Table 2).

Mutation rates are known to vary considerably across mammalian genomes [33]. It is conceivable that the ARs are biased towards regions of the genome with low mutation rates and that promoters are enriched in regions with high rates. Again, to explain the differences between primates and nonprimates, this bias would need to be more pronounced in primates. Gaffney and Keightley [34] have shown that the scale of these variations is on the order of 1 Mb, so that the substitution rates for two neutrally evolving regions of sequence are highly correlated if they lie within this distance of each other. We obtained intronic human versus chimpanzee rate estimates (see Materials and Methods) for 4,065 human genes for which we also had TSS defined promoter estimates. Thus, each of these promoter estimates was associated with a local estimate of the neutral substitution rate, the promoter and its corresponding intronic sequences being within 1 Mb of each other in every case. The mean intronic rate for human–chimpanzee comparisons was calculated as 0.0125 ± 0.0001, which is significantly lower than the rates observed in both the core promoter regions (0.0143 ± 0.0011) and upstream regions (0.0155 ± 0.0013) for these same genes. The intronic rate was also not significantly different from our genome-wide AR estimate (0.0127 ± 0.0001; Table 2), which suggests that neither was greatly influenced by regional variation in mutation rates across the genome. This demonstrates that we cannot explain the accelerated evolution of primate promoters by systematic biases in AR and promoter location with respect to large-scale (~1 Mb) fluctuations in mutation rate across the genome. However, the possibility remains that smaller islands of sequence around primate promoters have unusually high mutation rates, and, indeed, this explanation is consistent with the elevation in both substitutions and indels described in the previous section.

The molecular basis for this elevated promoter mutation rate may relate to the unusual chromatin structure in promoter regions. The higher order organisation of human chromosomes is still not well understood, particularly at high resolution. However, elegant work has shown that the chromatin structure within 1 kb of human TSSs is exceptionally open and accessible to allow the initiation and regulation of transcription [35]. In addition, recent work has suggested that regions of relatively open chromatin structure in the human genome are predisposed to higher levels of damage and mutation [36]. These observations are consistent with elevated mutation rates in promoter regions.

The third possibility to consider is that most primate promoters are subject to little purifying selection and so are evolving in a largely neutral manner. Combined with a small population of promoters subject to positive selection, this could drive the average substitution, insertion, and deletion rates above that calculated for AR. This is not as radical a proposal as it may first seem. Several recent papers [12–14] have suggested that because of the relatively small effective population size *(N_e_)* of humans and primates in general, this lineage is accumulating mildly deleterious mutations. It is argued that this has resulted in an almost complete loss of detectable constraint in regulatory regions, an effect which is pronounced in promoters [12]. We addressed this possibility by calculating a measure of the relative level of constraint *(C)* (see Materials and Methods) on a per-promoter basis and compared this to the level of coding sequence constraint for these same genes (Table S2). In total, we found that only 83 out of 11,478 (0.7%) analysed mouse promoters showed evidence for accelerated evolution (*C* < 0) in the rodent lineage (mouse versus rat). In comparison, 3,911 out of 6,404 human promoters (61.1%) were found to be evolving at a rate faster than that of ARs in the primate lineage (human versus chimpanzee). In contrast to the expectation that most promoters have been evolving neutrally in the primate lineage [12], we find that a clear majority are evolving faster than the AR rate. It is implausible that such a large effect could be principally due to widespread, sustained positive selection in the primate lineage. Nonetheless, we further investigated this possibility by considering the annotation and coding sequence evolution of the genes involved.

The functional annotation of these two sets of genes provides a striking contrast (Tables S3 and S4). The 83 mouse genes are significantly enriched for Gene Ontogeny (GO) terms concerned with host immunity (particularly natural-killer-cell-mediated functions) and apoptosis (Table S3). This profile of GO annotation is typical of genes than have been reported to be subject to positive selection on the basis of coding sequence *d*_N_/*d*_S_ measures [37]. Interestingly, in spite of the substantial change recorded in these mouse promoters, there was no evidence for positive selection in any of the associated coding sequences. Human genes possessing promoters with *C* < 0 showed no significant over-representation of GO terms typically associated with positively selected coding sequences, and were instead enriched for terms associated with basal metabolism, suggesting the over-representation of “housekeeping” genes in this list (Table S4). This enrichment applied to 61% of the promoters mapped to genes with GO annotation available. These observations suggest that, although the majority of human promoters are evolving more rapidly than AR, the dominant cause of accelerated evolution is not the action of positive selection.

We have explored several possible explanations for the apparent acceleration of evolution in primate promoters. We have been able to exclude artefacts due to sequence quality, large-scale variation in mutation rate, and widespread positive selection as principal causes. This leaves us with the baseline level of mutation in promoter regions. The simplest explanation would appear to be that there are elevated mutation rates in the immediate vicinity of primate promoters. This suggests that AR rates and local intron-based measures of *K* underestimate the neutral rate in primate promoters. More generally, this has implications for the identification of evolutionary constraint in promoters [12] and raises interesting questions as to the molecular basis of the elevated mutation rate.

### Mammalian Promoter Anatomy and Evolution

**Figure 4:**
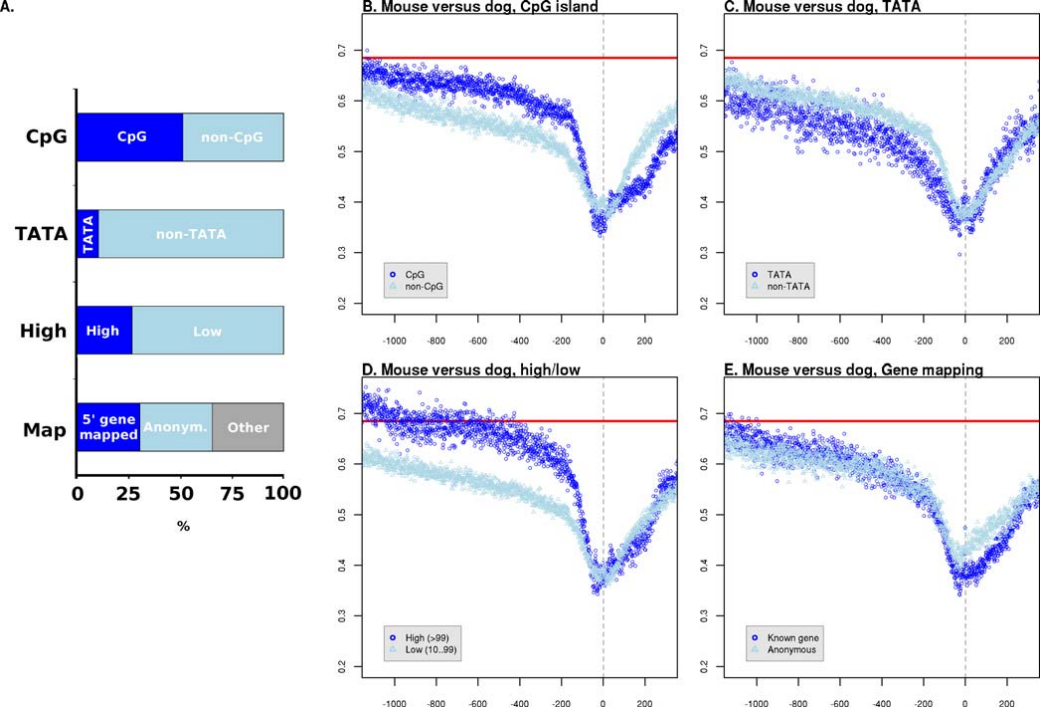
Patterns of Evolution in Promoter Subcategories (A) The percentage of all mouse TSSs assigned to each category. Dark blue shows the percentage assigned to the category annotated to the left, and light blue the reciprocal category (e.g., non-CpG is the reciprocal of CpG). The colour coding is consistent with (B–E). The “map” category refers to whether the TSS could be mapped to the annotated 5′-most end of a known protein-coding gene (dark blue), could not mapped to a gene (light blue), or maps internally to an annotated gene extent (grey). See Materials and Methods for details of category assignment. (B–E) Single nucleotide resolution estimates of substitution rates calculated from promoters assigned to the indicated categories. Only rates calculated from mouse–dog comparisons are shown. The 95% confidence intervals have been excluded for clarity. Red horizontal lines show *K* for ARs, nucleotide position is shown on the *x-*axis relative to the TSS at +1 (grey vertical line), and *K* is shown on the *y-*axis. Although there are three categories indicated for gene mapping in (A), only two are shown for clarity.

The large number of defined TSSs in this study allowed us to investigate the differences in evolution between distinct categories of promoters. For each of these categories we calculated *K* averaged across core promoter regions and upstream sequences (Tables 1 and 2). To compare the fine details of the conservation profiles between promoter types we also calculated rates at single nucleotide resolution (Figures 4, S2, and S3). If we disregard comparisons between promoters and ARs for the reasons discussed above, and concentrate on comparisons between promoter categories, we see a largely consistent picture regardless of lineage (Tables 1 and 2). TATA-box promoters tend to evolve more slowly in both core promoter and upstream regions than promoters lacking a TATA box (Tables 1 and 2). Promoters that can be mapped to a protein-coding gene evolve more slowly than those than cannot. Promoters represented by many tags, indicating a generally higher level of expression, are typically less constrained than those expressed at lower levels (Tables 1 and 2). But the higher resolution analysis shows that in the most proximal regions of the core promoter, constraint is actually greatest in the highly expressed genes (Figure 4D). This suggests that genes with lower levels or more restricted distribution of expression have more constrained regions upstream of the core promoter.

Several of the promoter categories considered were based on the distribution of CAGE tags around the TSS (see Materials and Methods) [20]. For the majority of these categories, we identified variable trends in both overall rates of evolution (Tables 1 and 2) and the higher resolution analyses (Figure 4 and data not shown) that were more clearly defined by assignment to other categories, such as whether the promoter contained a TATA box or was a CpG or non-CpG type. The exception to this was the single peak (SP) category, defined by a single dominant position within the cluster of tags defining the TSS. SP category promoters consistently exhibited the least constraint in core promoter regions, which is surprising as one may expect a tightly regulated TSS that can essentially only initiate at a single position to be associated with more, rather than less, evolutionary constraint.

As noted above, in general the relative constraint of promoter classes is consistent between lineages. There is one notable exception, that of CpG-island versus non-CpG-island promoters. Between closely related species such as human–chimpanzee, human–macaque, and mouse–rat, we find that CpG-island promoters are evolving at a slower rate than non-CpG-island promoters (Tables 1 and 2). In contrast, comparisons over larger phylogentic distances such as human–mouse, mouse–dog, and human–dog show the opposite pattern, CpG-island promoters evolving faster than non-CpG-island promoters (Tables 1 and 2). This observation cannot be explained by the accelerated evolution of primate promoters as it is not confined to the primate lineage and our observations of acceleration relative to the AR rate hold true across these promoter classes and species. That is, both CpG and non-CpG promoters are evolving faster than ARs in primates, whereas both types are generally evolving slower than ARs in the rodent lineage (Tables 1 and 2). Rather, this is evidence of a different heterotachy, which could be interpreted as the recent stability (that is, since the divergence of mouse from rat and human from macaque) of CpG islands following an earlier period of rapid evolution and instability when promoters could perhaps gain and lose CpG islands. The existence of such a period could explain the well-known differences in CpG-island architecture between rodent and primate genomes [30]. At this stage, however, this remains speculation and is likely to be a rewarding avenue for future investigation.

It would seem that promoter evolutionary rates in human and mouse genomes are mediated by promoter anatomy, so that different classes of promoter differ significantly in their substitution rates. These differences arise between classes defined by the possession or lack of a CpG island and between promoters with or without an associated protein-coding gene. Consistent, significant differences are also seen between classes defined by the spread of transcriptional activity around the primary TSS (the CAGE tag categories; see Materials and Methods). Such differences could be a confounding factor for studies of divergence based upon small numbers of promoters.

At the finer scale, there is a pronounced pattern of significant troughs and peaks in *K* within the 100 bp flanking the TSS in comparisons between all species (Figure S2), with three features most pronounced: a decrease in *K* 25–31 bp upstream of the TSS, a second decrease in *K* at 41–47 bp upstream, and a defined increase in *K* in the 2–3 nt immediately upstream of the TSS reference position. The consistency of these features across comparisons shows that the general pattern of selective constraint within promoters has been preserved between the mammalian lineages considered. It also demonstrates that we can detect evidence of selective constraint in primate promoters where it exists.

The region 25–31 bp upstream is spatially consistent with the TATA-box location (Figure S4). Upon removing all promoters with a predicted TATA box in this sequence range, most, but not all, of this pronounced dip in constraint is removed (Figure S4D). The residual signal is likely to represent functional TATA boxes not identified by the TRANSFAC matrix [38], a conclusion supported by the observation that the most commonly conserved residues in this range after TATA-box removal were still adenine and thymine, in contrast to cytosine and guanine being the most commonly conserved residues across the remainder of the core promoter region (data not shown). The second region of decreased *K* at 41–47 bp upstream of the TSS is also most prominent in TATA-box-containing promoters (Figure 4). There is no single dominant sequence motif at this position, suggesting that it may be a general spatial constraint for multiple factors. Interestingly, this site would be one helical turn of DNA upstream of the TATA box, an ideal location to mediate interactions with the TATA-binding protein and associated factors.

Surprisingly, although the TSS reference position is generally within a region of relatively low *K,* there is a modest but consistent peak in *K* 2–3 nt immediately upstream. This effect was found in all species comparisons and with further investigation was found in to be present in 5′ promoters but absent from internal promoters. The elevation of *K* at the TSS in 5′ promoters over internal promoters was statistically significant in all comparisons except mouse versus rat and human versus chimpanzee (three examples are shown in Figure S2). This effect may be a consequence of the strong compositional bias to guanine that is seen at the positions immediately adjacent to the TSS (P. Carninci, T. A. Sandelin, B. Lenhard, S. Katayama, K. Shimokawa, et al., unpublished data).

The relationship between selective constraint in coding sequence and neighbouring regulatory regions is poorly understood, but closer examination of the present data suggests that they are seldom coupled in either the rodent or primate lineages. Table S2 shows that in the rodent lineage, as expected, coding sequence is more constrained than core promoter sequences, reflecting the low density of constrained sites in regulatory sequences relative to coding sequence. Across all mouse promoters, the level of relative constraint *(C)* achieved in the rodent lineage (0.754) is 92% of that in coding sequence (0.816). In contrast, the relative constraint in human core promoters (−0.101) differs radically from that measured in coding sequences (0.294). The only human core promoters with *C* significantly greater than zero are those lacking CpG islands (the other human types in Table S2 meeting this description overlap this category), and these promoters seem to be associated with the most constrained primate coding sequences. Only the mouse “high” category (representing relatively highly expressed genes) shows a significant, though modest, correlation between constraint in coding and promoter sequence (Table S2). (Note that the other Table S2 types showing a significant correlation are subsets of this category.) No such correlation is evident across mouse core promoters as a whole.

### Conclusions

In summary, evolutionary rates vary both on a fine scale within mammalian promoters and also between different functional classes of promoters. What may be thought of as “generic” promoters situated at the 5′ ends of protein-coding genes evolve quite differently from other classes, particularly those not associated with such genes. Similarly, anatomical categories based upon the presence of CpG islands and the TATA-box motif display characteristic differences. The rate of promoter evolution relative to other sequences also varies across lineages. For instance, we have found evidence for increased rates of change in primate promoters relative to neutral control sequences expected to reflect the background, genomic mutation rates. This increase is seen across different classes of mutation, including substitutions and micro-indel events, and suggests distinct peculiarities in the spectrum of mutations suffered by primate promoters. Keightley et al. [12] reported that *K* in 1,000 upstream sequences was not significantly different from that seen in a putatively neutral dataset of intronic sequences, and concluded that there had been a catastrophic loss of constraint in primate promoters. In contrast, with a larger dataset and based upon experimentally defined TSSs, we find that *K* in primate promoters consistently and significantly exceeds that seen in near-neutral controls. This accelerated evolution is not explicable by a relaxation of selective constraint alone, and we find no evidence that it is attributable to sequencing error or widespread positive selection. Increased mutation rates at primate promoters would appear to be sufficient to explain the acceleration seen, though given the present data we cannot exclude an accompanying reduction in the efficiency of purifying selection. In any case, it would appear that evolution within core promoters has been relatively rapid for perhaps 25 million years of primate evolution and that this may be a distinctive characteristic of our mammalian order. These results have implications for the discovery of regulatory elements within promoters using comparative genomics, particularly where such elements are defined using comparisons among primate species [39]. Elements that are only weakly constrained relative to the neutral substitution rate are likely to be indiscernible even with large numbers of primate species. On the other hand, where primate-specific elements are strongly constrained, as some are [39], an unexpectedly high substitution rate in flanking regions may aid in their detection.

## Materials and Methods

### TSS alignments.

TSSs were derived from mouse and human FANTOM3 CAGE-tag clusters composed of ten or more tags; a more conservative set of TSSs derived from 100 or more tag clusters was also analysed [20]. The reference position, designated +1, for a TSS was defined by the genome-aligned position of the modal tag from the tag cluster [20]. We defined a single orthologous genomic segment in target genomes using the University of California Santa Cruz (UCSC) Genome Browser (http://genome.ucsc.edu) comparative alignments [40], first using “nets” to define a single alignment “chain” that either aligned directly with the TSS +1 position or represented the lowest level chain that captured the +1 position in an alignment gap. Using this chain, positions corresponding to −1,200 through to +400 relative to the +1 position in the reference sequence were mapped onto the target genome. If no chain could be defined, we considered it unalignable in that target genome and treated it as missing data in subsequent analyses. Where these outer coordinates could not be mapped precisely, we assigned them to the nearest aligned position in the target genome. These orthologous genomic intervals were extracted and multiple sequence global alignments produced with MLAGAN (version 1.21) [41], making use of soft-masking for interspersed repetitive elements and low-complexity sequence (guided by UCSC Genome Browser annotation [40]). The alignment guide tree was based on the topology used by Margulies et al. [42]. Mouse TSS analyses were based on alignments of the mm5, hg17, rn3, and canFam1 (UCSC Genome Browser nomenclature) assemblies. Human TSS alignments were between the hg17, panTro1, rheMac1, mm6, and canFam1 assemblies. Although the sequence range −1,200 to +400 of the reference sequence and orthologous extents from other genomes were aligned, we only considered regions of the alignments corresponding to −1,100 to +300 of the reference sequence, or subsets of that range, in analyses to avoid alignment edge effects.

Promoters with sufficient data (those with TSSs supported by more than 100 tag clusters) were categorised according to the four categories of TSSs previously discovered in the CAGE data itself (multimodal [MU], broad [BR], broad with a dominant peak [PB], and SP). Briefly, these four categories are based upon the distribution of CAGE tags around the predicted TSS (P. Carninci, T. A. Sandelin, B. Lenhard, S. Katayama, K. Shimokawa, et al., unpublished data): the SP type shows a distinct TSS and is associated with the presence of a TATA box, whereas the other three types show broader distributions around the preferred TSS and are often associated with predicted CpG islands. Other categories were constructed according to whether promoters possessed a predicted CpG island (taken from UCSC Genome Browser annotation [40]) or a TATA box (predicted as matches to the TRANSFAC profileV$TATA_01 [38] attaining a minimum of 75% of the maximum possible weight matrix score and located within 50 bp of the TSS). Those promoters with TSSs supported by 100 or more tags (“high”) and those supported by fewer than 100 tags (“low”) were also examined, as a simple way to examine rate differences between promoters associated with relatively high and low rates of transcription.

### Substitution rate calculations.

Pairwise substitution rates (and 95% confidence intervals) for the sequences in each alignment were estimated using the REV model in PAML 3.14 [43], as recommended by Yap and Pachter [44]. All TSS alignments were masked for CpG islands and simple repeats (based upon UCSC Genome Browser annotation for the human hg17 and mouse mm5 genomes [40]) before rate estimates were made, as such regions are known to evolve by mechanisms other than point mutation. Both high- and low-resolution estimates of substitution rates were calculated. For each alignment, divergence was measured for the entire upstream and downstream regions as well as in the 200-bp core promoter region immediately upstream of the TSS; these constitute low-resolution estimates. To ensure the accuracy and statistical strength of these estimates, we removed all alignments containing fewer than 100 aligned nucleotides. For high-resolution estimates (providing up to 1 bp resolution), the following strategy was followed. All TSS alignments under study were compressed by removing columns containing a gap in the reference (human hg17 and mouse mm5) sequence. Then concatenated alignments were constructed for each position across the 1,400-bp alignments; for example, all alignment columns corresponding to basepair 1 from all TSS alignments were concatenated to give a single alignment *n* bp long, where *n* is the number of alignments under study. The result was 1,400 alignments that were used sequentially as input to PAML to give a detailed picture of substitution rates across the TSS alignments. All substitution rate estimates are given with the 95% confidence intervals calculated using PAML standard errors.

### Ancient repeat sequences.

We also extracted a large number of UCSC Genome Browser hg17 orthologous regions corresponding to ARs (14,460 regions encompassing 3,443,541 bp). These represent a randomly selected 10% of the ARs that were identified. AR was defined as by Gibbs et al. [7] as interspersed repeats, from the same RepeatMasker subfamily, in conserved orientation shared between mouse and human. We also required that each repeat have one or more nucleotides aligned in each of human, chimpanzee, macaque, mouse, rat, and dog. These regions were aligned using MLAGAN as before, but with soft-masking of only low-complexity sequence and not interspersed repeats, to provide an approximately neutrally evolving set of alignments. These alignments were then compressed and concatenated as above to provide a single alignment as input to PAML (REV model) and consequently an estimate of the neutral substitution rate for each species pair. Again, all substitution rate estimates are given with the 95% confidence intervals calculated using PAML standard error estimates. The relative level of constraint *(C)* in promoters was calculated as the promoter *K* divided by that of ARs and then subtracted from one, so that increasing *C* suggests greater selective constraint. All alignments analysed here are available at http://www.hgu.mrc.ac.uk/Users/Colin.Semple/lab_data.html. Displays and downloads of all FANTOM3 CAGE data are also publicly available (http://fantom3.gsc.riken.jp).

Mean repeat densities and lengths were calculated using UCSC RepeatMasker [45] annotation for the mouse and human genomic sequence assemblies. Because of the large number of subclasses and families, only the main repeat class densities were examined. Note that the “other” class contains repeats that are currently unclassified and the “unknown” class is used for the small number of known repeats that have not been assigned a class. All absolute repeat densities estimated are likely to be overestimates because of fragmentary elements that were counted more than once by RepeatMasker, so conclusions are drawn only on the basis of relative densities, using comparisons to genome-wide means.

### TSS-to-gene mapping and GO analysis.

Predicted orthologous gene pairs and their corresponding *d*_N_ and *d*_S_ estimates were extracted from Ensembl Human (release 31.35d) and Ensembl Mouse (release 31.33g) [46]. TSSs were associated with Ensembl genes if they mapped within 500 bp of, and in conserved orientation with, the 5′ end of the gene (these TSSs were designated 5′ promoters), or otherwise within the span of an Ensembl gene (“internal” promoters). Such internal promoters are expected, since most genes appear to contain alternative TSSs, and transcripts can also originate from TSSs within 3′ untranslated regions [20]. The remaining TSSs that failed to map within Ensembl genes and their 5′ flanking sequences were assigned to the “no gene” class. Intronic rate estimates for orthologous human and chimpanzee gene pairs were obtained from a recent study and were calculated using PAML (REV model) [32]. Statistical analyses of GO term over-representation among genes consisted of hypergeometric tests (equivalent to Fisher's exact tests) with a false discovery rate correction, and were performed using Cytoscape [47] with the BiNGO plug-in [48].

### Insertion and deletion rates.

Three-way alignments representing two relatively closely related species (e.g., human and chimpanzee) and an outgroup species (e.g., macaque) were extracted from the multiple sequence MLAGAN alignments, and fully gapped columns were removed. Micro-insertions and -deletions (<11 nucleotides) were identified, and the lineage and direction of change was resolved as described previously [26]. We only considered indels that were flanked by eight ungapped alignment columns and that did not overlap any other alignment gaps. The indel rate was calculated as the number of events (insertion or deletion) divided by the number of ungapped alignment columns. Promoter regions were analysed as consecutive 100-nt windows, based on the coordinates of the TSS reference sequence (e.g., human). The final rate estimate and 95% confidence intervals were derived from the mean of 1,000 bootstrap samplings (with replacement) from a population of equivalent window positions (e.g., windows from each promoter corresponding to nucleotides one to 100 of the aligned reference sequence). Rate estimates for repetitive sequences were calculated as above, but with a single window encompassing the entire repeat sequence alignment. As these measures could—for comparisons between closely related species—be dominated by sequence errors in lower quality genome sequences, we only considered indel rates for terminal branches of species with high-quality genome sequence (human and mouse).

## Supporting Information

Figure S1 High-Resolution Pairwise Transversion and Transition Rates Based on human versus macaque comparisons. Green shows transitions and blue transversions. Horizontal lines show rates calculated from ARs. Error bars are excluded for clarity.
(36 KB PDF)

Figure S2 High-Resolution Pairwise Substitution Rate Estimates in the Immediate Vicinity of the TSS for All Human- and Mouse-Based Alignments (A–C) Mouse-based alignments.
(D–G) Human based alignments.
The *x-*axis shows position relative to the TSS reference position, indicated by a vertical grey line at +1. Error bars show 95% confidence intervals.
(70 KB PDF)

Figure S3 Pairwise Substitution Rate Estimates in the Immediate Vicinity of the TSS for 5′ and Internal Promoters Results are displayed for 5′ promoters (5p) in red and for internal promoters (int) in blue, for three different species comparisons. In all data the TSS is at position +1, indicated by a grey vertical line. Error bars show 95% confidence intervals.
(38 KB PDF)

Figure S4 Influence of TATA Boxes on Promoter Evolutionary Rate Profiles (A) The 5′-most position of matches to a TATA-box profile across human promoter regions. The *x*-axis indicates position, with the TSS at +1; the *y*-axis shows the number of promoters with a significant match to the TATA-box profile (see Materials and Methods).
(B) The same data as in (A) but focussed in on the region immediately around the TSS. A clear and sharp peak is evident at −33 to −27, showing great consistency in the spacing between the TATA box and the dominant TSS.
(C) The substitution rate calculated per nucleotide across human promoters based on human–mouse alignment. The subset of promoters that match the TATA-box profile in the nucleotide range −33 to −27 are shown in blue, and those without a TATA-box match are shown in red. Error bars indicate 95% confidence intervals.
(D) The same data as in (C) but focussed in on the region immediately around the TSS. The significant reduction in substitution rate around −30 is confined to sequences with a TATA-box match.
(34 KB PDF)

Table S1 Substitution Rate Estimates for Human Promoters after Masking for CpG Substitution Effects All nucleotides preceding a cytosine or following a guanine in human sequence were masked in the alignments prior to substitution rates being calculated, as described in Materials and Methods. The 95% confidence intervals are shown in parentheses.
(13 KB PDF)

Table S2 Relative Selective Constraint in Mouse and Human Coding and Core Promoter Sequences The table shows the number of promoters analysed *(n),* the mean selective constraint for flanking coding sequence (1 − *d*_N_/*d*_S_; 95% confidence intervals in parentheses), the mean selective constraint in promoters (*C,* 95% confidence intervals in parentheses), and the Pearson's correlation coefficient between 1 − *d*_N_/*d*_S_ and *C* (*r;* **, *p* < 0.01; ***, *p* < 0.001). Estimates are given for all promoters considered together (“all”) and various categories of promoters: the promoter classes discovered in the CAGE data (MU, BR, PB, and SP), promoters possessing versus not possessing CpG islands or TATA boxes, promoters with TSSs supported by 100 or more tags (“high”) and fewer than 100 tags (“low”), and promoters at 5′ ends of genes (5p), internal (int) to genes, or not mapped to genes (“no gene”).
(25 KB PDF)

Table S3 Significantly Over-Represented GO Annotation Terms within Mouse Genes Possessing Rapidly Evolving Promoters with *C* < 0 GO biological process ID numbers and descriptions are given together with the number of genes under scrutiny possessing the GO term *(n),* the total number of mouse genes annotated with the GO term (“total”), and the corrected *p*-value calculated for the enrichment of the GO term.
(22 KB PDF)

Table S4 Significantly Over-Represented GO Annotation Terms within Human Genes Possessing Rapidly Evolving Promoters with *C* < 0 GO biological process ID numbers and descriptions are given together with the number of genes under scrutiny possessing the GO term *(n),* the total number of human genes annotated with the GO term (“total”), and the corrected *p*-value calculated for the enrichment of the GO term.
(75 KB PDF)

## Abbreviations

AR: ancient repeat sequences
GO: Gene Ontogeny
indel: insertion or deletion
nt: nucleotide(s)
SP: single peak
TFBS: transcription factor binding site
TSS: transcription start site
UCSC: University of California Santa Cruz
