# Supplementary figures and images for: Heterotachy in Mammalian Promoter Evolution
Source: PLoS Genet. 2006 Apr 28;2(4):e30. doi: 10.1371/journal.pgen.0020030 (PMC1449885; doi:10.1371/journal.pgen.0020030)

**Figure S1**

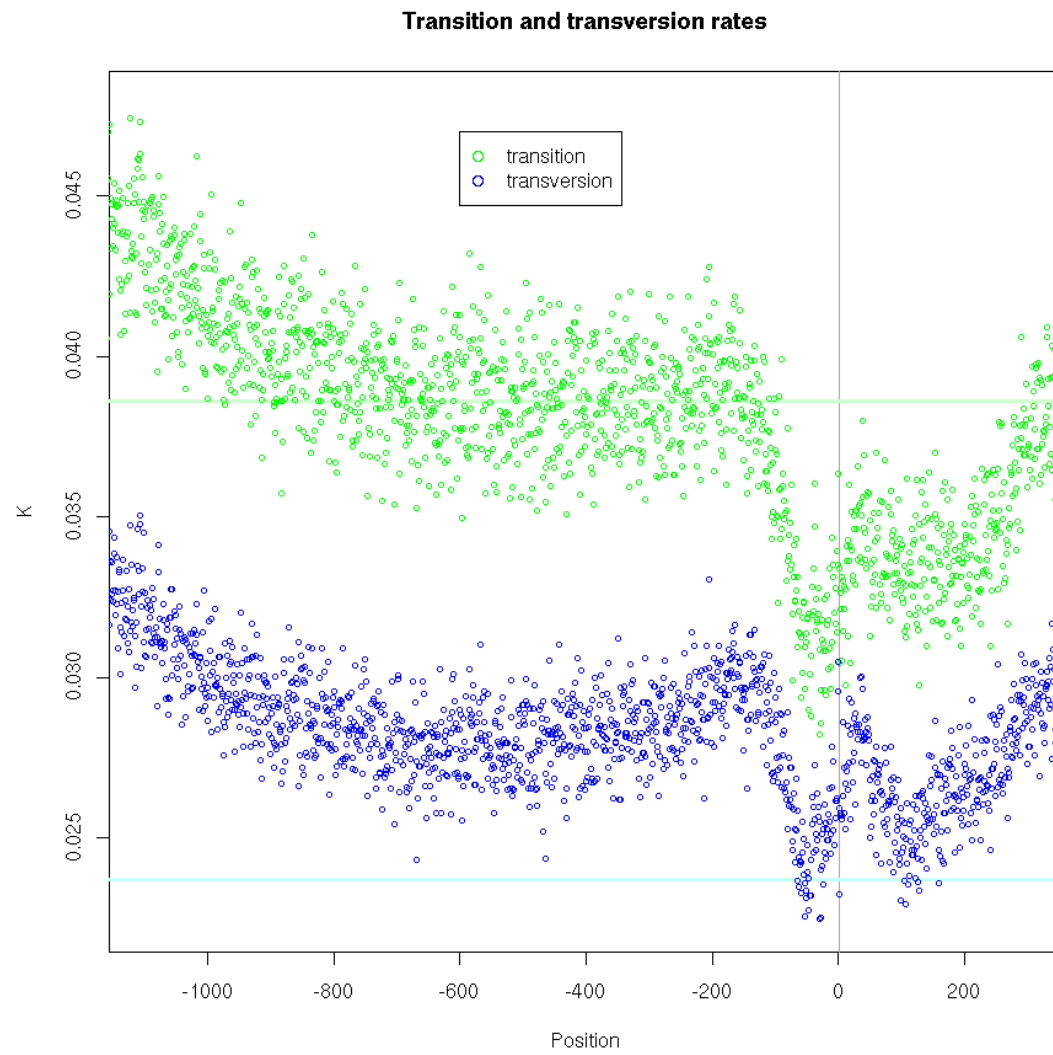

Supplement: Figure S1 — Based on human versus macaque comparisons. Green shows transitions and blue transversions. Horizontal lines show rates calculated from ARs. Error bars are excluded for clarity. (36 KB PDF) [file pgen.0020030.sg001.pdf]

Figure S2

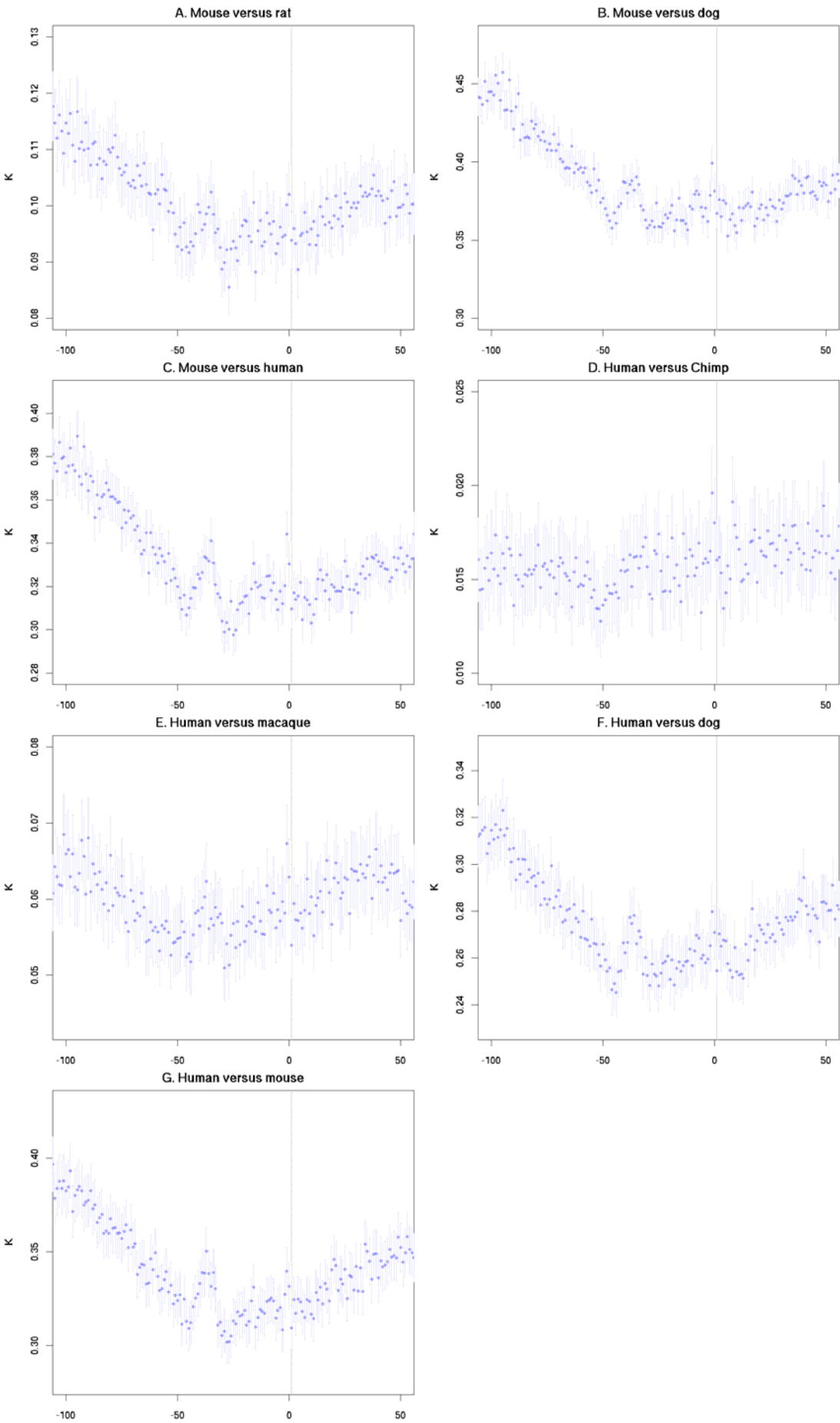

Supplement: Figure S2 — (A–C) Mouse-based alignments. (D–G) Human based alignments. The x-axis shows position relative to the TSS reference position, indicated by a vertical grey line at +1. Error bars show 95% confidence intervals. (70 KB PDF) [file pgen.0020030.sg002.pdf]

Figure S3

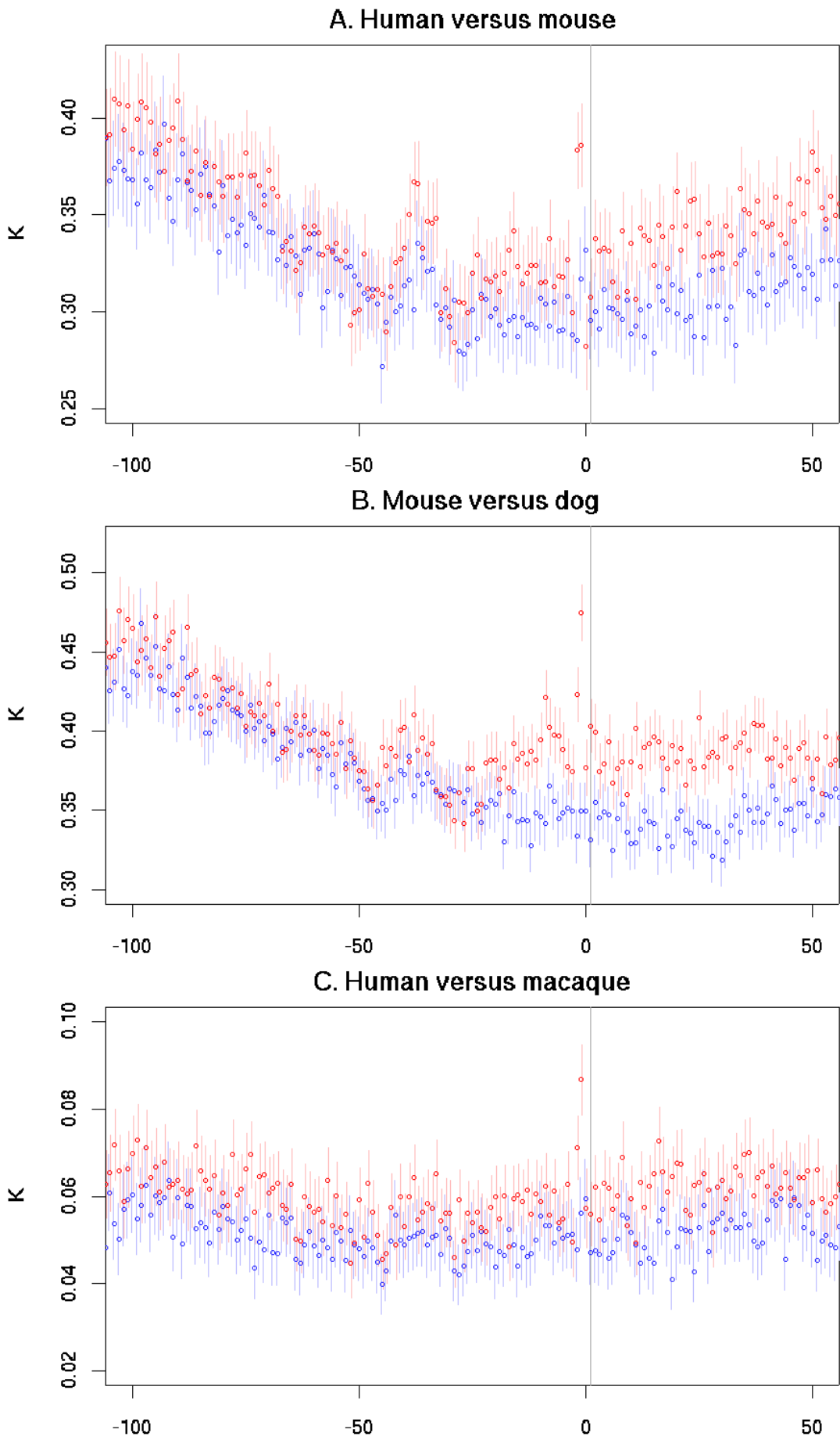

Supplement: Figure S3 — Results are displayed for 5′ promoters (5p) in red and for internal promoters (int) in blue, for three different species comparisons. In all data the TSS is at position +1, indicated by a grey vertical line. Error bars show 95% confidence intervals. (38 KB PDF) [file pgen.0020030.sg003.pdf]

**Figure S4**

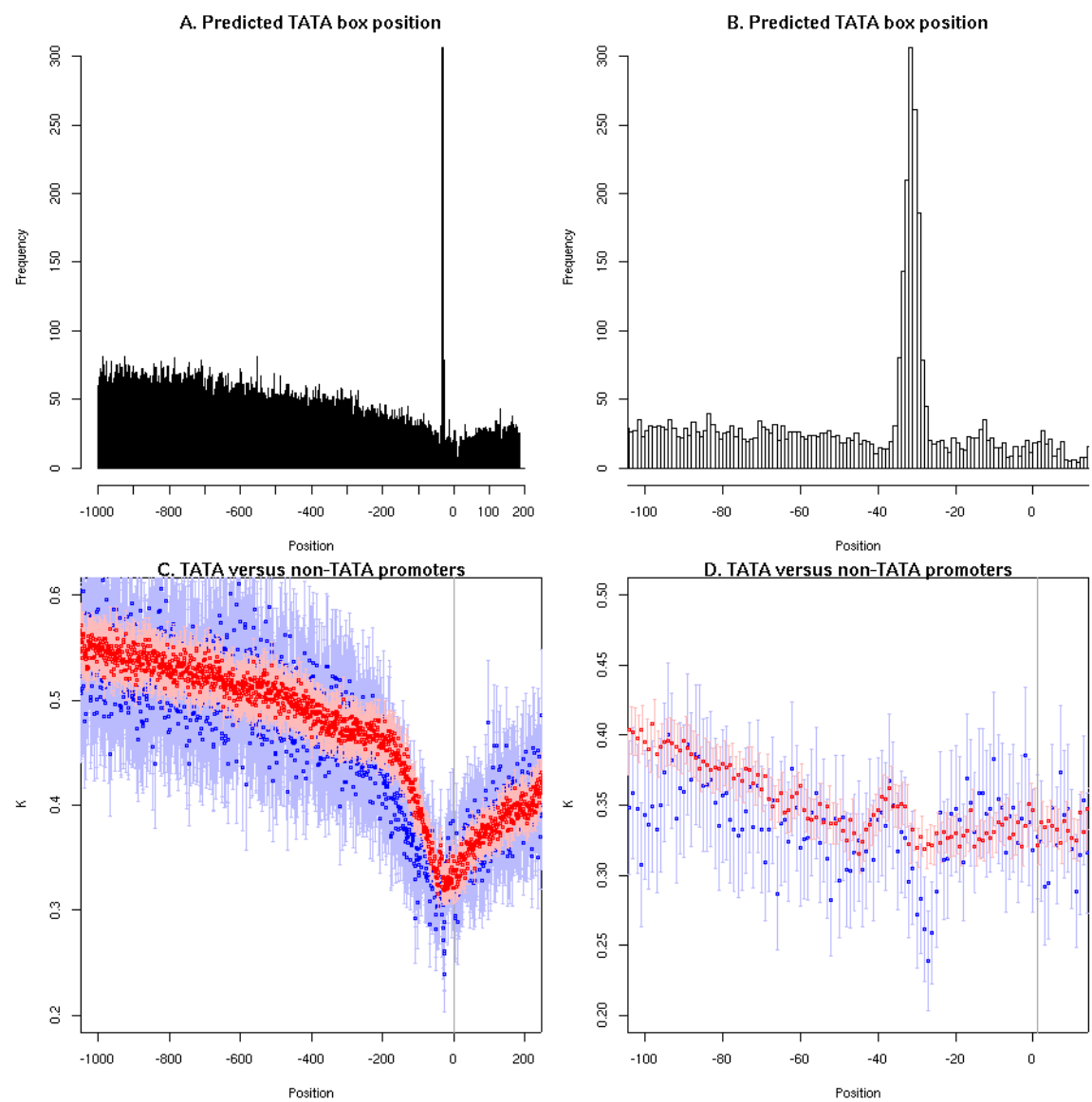

Supplement: Figure S4 — (A) The 5′-most position of matches to a TATA-box profile across human promoter regions. The x-axis indicates position, with the TSS at +1; the y-axis shows the number of promoters with a significant match to the TATA-box profile (see Materials and Methods). (B) The same data as in (A) but focussed in on the region immediately around the TSS. A clear and sharp peak is evident at −33 to −27, showing great consistency in the spacing between the TATA box and the dominant TSS. (C) The substitution rate calculated per nucleotide across human promoters based on human–mouse alignment. The subset of promoters that match the TATA-box profile in the nucleotide range −33 to −27 are shown in blue, and those without a TATA-box match are shown in red. Error bars indicate 95% confidence intervals. (D) The same data as in (C) but focussed in on the region immediately around the TSS. The significant reduction in substitution rate around −30 is confined to sequences with a TATA-box match. (34 KB PDF) [file pgen.0020030.sg004.pdf]
