# Supplementary material for: Heterotachy in Mammalian Promoter Evolution
Source: PLoS Genet. 2006 Apr 28;2(4):e30. doi: 10.1371/journal.pgen.0020030 (PMC1449885; doi:10.1371/journal.pgen.0020030)
Supplement: Table S1 — All nucleotides preceding a cytosine or following a guanine in human sequence were masked in the alignments prior to substitution rates being calculated, as described in Materials and Methods. The 95% confidence intervals are shown in parentheses. (13 KB PDF) [file pgen.0020030.st001.pdf]

Table S2

Species	AR	Core promoter	Upstream sequence
**Human:Chimp**	0.01347 (0.00015)	0.017172 (0.001412)	0.019306 (0.000687)
**Human:Macaque**	0.06399 (0.00041)	0.069287 (0.002538)	0.085674 (0.001424)
**Human:Mouse**	0.57039 (0.0015)	0.422786 (0.007153)	0.542568 (0.002447)
**Human:Dog**	0.39785 (0.0013)	0.335408 (0.006283)	0.452756 (0.002448)
