# Supplementary material for: Heterotachy in Mammalian Promoter Evolution
Source: PLoS Genet. 2006 Apr 28;2(4):e30. doi: 10.1371/journal.pgen.0020030 (PMC1449885; doi:10.1371/journal.pgen.0020030)
Supplement: Table S2 — The table shows the number of promoters analysed (n), the mean selective constraint for flanking coding sequence (1 − d N/d S; 95% confidence intervals in parentheses), the mean selective constraint in promoters (C, 95% confidence intervals in parentheses), and the Pearson's correlation coefficient between 1 − d N/d S and C (r; **, p < 0.01; ***, p < 0.001). Estimates are given for all promoters considered together (“all”) and various categories of promoters: the promoter classes discovered in the CAGE data (MU, BR, PB, and SP), promoters possessing versus not possessing CpG islands or TATA boxes, promoters with TSSs supported by 100 or more tags (“high”) and fewer than 100 tags (“low”), and promoters at 5′ ends of genes (5p), internal (int) to genes, or not mapped to genes (“no gene”). (25 KB PDF) [file pgen.0020030.st002.pdf]

Table S1

**Type**	**n**	**1-(dN/dS)**	**C**	**r**
**Mouse**				
MU	613	0.867 (0.011)	0.760 (0.013)	0.155***
BR	952	0.874 (0.008)	0.740 (0.009)	0.081**
PB	688	0.869 (0.009)	0.733 (0.015)	0.106**
SP	637	0.846 (0.010)	0.751 (0.013)	0.104**
CpG	4724	0.843 (0.047)	0.769 (0.005)	0.000
Non-CpG	6413	0.802 (0.048)	0.748 (0.005)	-0.006
TATA	1284	0.832 (0.019)	0.752 (0.005)	-0.007
Non-TATA	9853	0.822 (0.037)	0.754 (0.004)	-0.003
High	2909	0.831 (0.035)	0.752 (0.004)	0.104***
Low	8228	0.812 (0.038)	0.758 (0.004)	-0.006
5p	4890	0.833 (0.029)	0.759 (0.004)	-0.004
Int	6247	0.835 (0.025)	0.749 (0.004)	-0.002
All	11478	0.816 (0.033)	0.754 (0.004)	-0.001
**Human**				
MU	173	0.264 (0.045)	0.148 (0.129)	-0.025
BR	271	0.218 (0.033)	-0.446 (0.682)	-0.071
PB	189	0.287 (0.053)	-0.192 (0.204)	-0.207
SP	179	0.278 (0.053)	-0.390 (0.559)	0.038
CpG	2519	0.286 (0.015)	-0.177 (0.125)	-0.050
Non-CpG	576	0.330 (0.032)	0.232 (0.079)	-0.081
TATA	234	0.344 (0.060)	0.191 (0.108)	-0.004
Non-TATA	2861	0.290 (0.014)	-0.125 (0.111)	-0.051
High	812	0.257 (0.023)	-0.248 (0.265)	-0.040
Low	2283	0.307 (0.017)	-0.049 (0.102)	-0.056
5p	3095	0.294 (0.014)	-0.101 (0.103)	-0.047
Int	0	0 (0)	0 (0)	0
All	3095	0.294 (0.014)	-0.101 (0.103)	-0.047
