# Supplementary material for: Heterotachy in Mammalian Promoter Evolution
Source: PLoS Genet. 2006 Apr 28;2(4):e30. doi: 10.1371/journal.pgen.0020030 (PMC1449885; doi:10.1371/journal.pgen.0020030)
Supplement: Table S3 — GO biological process ID numbers and descriptions are given together with the number of genes under scrutiny possessing the GO term (n), the total number of mouse genes annotated with the GO term (“total”), and the corrected p-value calculated for the enrichment of the GO term. (22 KB PDF) [file pgen.0020030.st003.pdf]

Table S3

GO-ID	p-value	n	Total	Description
42269	7.1173E-5	3	5	regulation of natural killer cell mediated cytotoxicity
42267	7.1173E-5	3	5	natural killer cell mediated cytotoxicity
31341	1.2387E-4	3	7	regulation of cell killing
1910	1.2387E-4	3	7	regulation of immune cell mediated cytotoxicity
1909	3.8506E-4	3	11	immune cell mediated cytotoxicity
1906	3.8506E-4	3	11	cell killing
42981	5.2382E-4	7	208	regulation of apoptosis
43067	5.2382E-4	7	211	regulation of programmed cell death
43065	9.2493E-4	5	96	positive regulation of apoptosis
43068	9.2493E-4	5	97	positive regulation of programmed cell death
44419	1.2067E-3	3	19	interaction between organisms
31342	1.6414E-3	2	4	negative regulation of cell killing
1911	1.6414E-3	2	4	negative regulation of immune cell mediated cytotoxicity
45953	1.6414E-3	2	4	negative regulation of natural killer cell mediated cytotoxicity
6917	4.3626E-3	4	81	induction of apoptosis
12502	4.3626E-3	4	81	induction of programmed cell death
6915	7.7732E-3	7	367	apoptosis
12501	8.0852E-3	7	373	programmed cell death
8219	1.2262E-2	7	404	cell death
16265	1.2877E-2	7	411	death
45954	4.8484E-2	1	1	positive regulation of natural killer cell mediated cytotoxicity
