# Supplementary material for: Heterotachy in Mammalian Promoter Evolution
Source: PLoS Genet. 2006 Apr 28;2(4):e30. doi: 10.1371/journal.pgen.0020030 (PMC1449885; doi:10.1371/journal.pgen.0020030)
Supplement: Table S4 — GO biological process ID numbers and descriptions are given together with the number of genes under scrutiny possessing the GO term (n), the total number of human genes annotated with the GO term (“total”), and the corrected p-value calculated for the enrichment of the GO term. (75 KB PDF) [file pgen.0020030.st004.pdf]

Table S4

GO-ID	p-value	n	Total	Description
8151	7.7360E-21	2212	9276	cellular physiological process
5622	1.4058E-20	1716	6910	intracellular
44237	2.8411E-19	1665	6712	cellular metabolism
8152	6.0287E-19	1752	7132	metabolism
44238	4.2413E-16	1586	6442	primary metabolism
43227	2.2560E-15	1264	4993	membrane-bound organelle
43231	2.2560E-15	1264	4993	intracellular membrane-bound organelle
3824	5.3170E-14	1233	4895	catalytic activity
43226	2.9497E-13	1418	5764	organelle
43229	2.9497E-13	1418	5764	intracellular organelle
5737	3.2041E-12	758	2849	cytoplasm
43170	1.9956E-11	850	3272	macromolecule metabolism
44260	2.9870E-11	807	3090	cellular macromolecule metabolism
19538	1.3435E-10	761	2909	protein metabolism
44267	4.5970E-10	752	2886	cellular protein metabolism
5634	4.8937E-9	895	3548	nucleus
6464	8.1933E-8	424	1544	protein modification
7049	1.3856E-6	208	687	cell cycle
166	1.4999E-6	425	1582	nucleotide binding
3723	1.8085E-6	163	514	RNA binding
17076	2.4375E-6	417	1555	purine nucleotide binding
43283	1.1196E-5	431	1634	biopolymer metabolism
74	1.2196E-5	135	420	regulation of cell cycle
6139	1.5698E-5	750	3036	nucleobase, nucleoside, nucleotide and nucleic acid metabolism
5739	1.7091E-5	186	622	mitochondrion
43234	1.8146E-5	426	1621	protein complex
16568	1.8225E-5	48	111	chromatin modification
44249	1.8839E-5	271	969	cellular biosynthesis
9058	2.7932E-5	300	1094	biosynthesis
16772	4.4921E-5	259	929	transferase activity, transferring phosphorus-containing groups
16740	5.7399E-5	410	1570	transferase activity
45308	5.7399E-5	629	2526	protein binding
6259	7.9723E-5	172	581	DNA metabolism
5524	1.0124E-4	321	1199	ATP binding
30554	1.2425E-4	328	1232	adenyl nucleotide binding
6796	1.2425E-4	223	793	phosphate metabolism
6793	1.2425E-4	223	793	phosphorus metabolism
16043	1.7941E-4	251	913	cell organization and biogenesis
16874	2.5111E-4	156	528	ligase activity
6996	2.5820E-4	213	760	organelle organization and biogenesis
46907	3.0320E-4	154	522	intracellular transport
8371	3.7837E-4	63	175	obsolete biological process
16288	4.9354E-4	57	155	cytokinesis
50789	5.4327E-4	769	3199	regulation of biological process
9117	6.0307E-4	67	192	nucleotide metabolism

5488	6.6941E-4	1933	8613	binding
6886	8.2712E-4	101	322	intracellular protein transport
16773	1.1613E-3	186	666	phosphotransferase activity, alcohol group as acceptor
6416	1.5006E-3	158	554	protein biosynthesis
51186	1.5393E-3	57	161	cofactor metabolism
45184	1.5393E-3	144	498	establishment of protein localization
16407	1.5393E-3	24	50	acetyltransferase activity
50794	1.5393E-3	701	2919	regulation of cellular process
50791	1.5393E-3	707	2946	regulation of physiological process
4674	1.7012E-3	125	423	protein serine/threonine kinase activity
9059	1.7080E-3	173	618	macromolecule biosynthesis
15031	1.7630E-3	143	496	protein transport
16301	1.7999E-3	218	806	kinase activity
8104	2.3109E-3	146	511	protein localization
6974	2.3442E-3	70	212	response to DNA damage stimulus
4672	2.7091E-3	159	566	protein kinase activity
6281	3.4731E-3	64	192	DNA repair
9719	3.4756E-3	73	226	response to endogenous stimulus
43037	3.5227E-3	58	170	translation
8134	4.0054E-3	87	281	transcription factor binding
16879	4.0586E-3	122	420	ligase activity, forming carbon-nitrogen bonds
31090	4.0598E-3	114	388	organelle membrane
6366	4.1554E-3	133	465	transcription from RNA polymerase II promoter
51244	4.3644E-3	663	2776	regulation of cellular physiological process
16310	4.4423E-3	176	643	phosphorylation
3676	5.2019E-3	739	3124	nucleic acid binding
6446	5.4918E-3	16	30	regulation of translational initiation
278	7.2076E-3	78	251	mitotic cell cycle
16817	8.3121E-3	146	526	hydrolase activity, acting on acid anhydrides
17111	8.9413E-3	138	494	nucleoside-triphosphatase activity
16818	8.9413E-3	145	523	hydrolase activity, acting on acid anhydrides, in phosphorus-containing anhydrides
9889	8.9413E-3	43	121	regulation of biosynthesis
7001	8.9413E-3	78	253	chromosome organization and biogenesis (sensu Eukaryota)
8361	9.5810E-3	47	136	regulation of cell size
16049	9.5810E-3	47	136	cell growth
3712	9.6167E-3	76	246	transcription cofactor activity
46483	1.0079E-2	23	53	heterocycle metabolism
16462	1.0079E-2	144	521	pyrophosphatase activity
16787	1.0378E-2	473	1948	hydrolase activity
6325	1.0576E-2	68	216	establishment and/or maintenance of chromatin architecture
6323	1.0889E-2	70	224	DNA packaging
51169	1.0940E-2	37	101	nuclear transport

49	1.1024E-2	9	13	tRNA binding
30529	1.1216E-2	99	339	ribonucleoprotein complex
45182	1.1611E-2	38	105	translation regulator activity
6605	1.1611E-2	46	134	protein targeting
6454	1.1611E-2	24	57	translational initiation
8135	1.5979E-2	37	103	translation factor activity, nucleic acid binding
6417	1.6435E-2	40	114	regulation of protein biosynthesis
4177	1.7368E-2	16	33	aminopeptidase activity
3743	1.8571E-2	25	62	translation initiation factor activity
16569	1.9599E-2	11	19	covalent chromatin modification
16570	1.9599E-2	11	19	histone modification
43122	1.9977E-2	31	83	regulation of I-kappaB kinase/NF-kappaB cascade
6913	1.9977E-2	38	108	nucleocytoplasmic transport
43123	2.2158E-2	30	80	positive regulation of I-kappaB kinase/NF-kappaB cascade
6512	2.3560E-2	150	558	ubiquitin cycle
6091	2.3986E-2	158	592	generation of precursor metabolites and energy
9165	2.4297E-2	45	135	nucleotide biosynthesis
1558	2.6058E-2	37	106	regulation of cell growth
16779	2.6058E-2	40	117	nucleotidyltransferase activity
9260	2.6058E-2	30	81	ribonucleotide biosynthesis
6445	2.6058E-2	28	74	regulation of translation
51276	2.6058E-2	89	308	chromosome organization and biogenesis
15078	2.6058E-2	48	147	hydrogen ion transporter activity
8415	2.6450E-2	41	121	acyltransferase activity
16747	2.6450E-2	41	121	transferase activity, transferring groups other than amino-acyl groups
9967	2.7031E-2	33	92	positive regulation of signal transduction
16410	2.7031E-2	19	44	N-acyltransferase activity
5654	2.7546E-2	68	225	nucleoplasm
16881	2.8920E-2	108	387	acid-amino acid ligase activity
16485	3.2232E-2	12	23	protein processing
5525	3.2546E-2	94	331	GTP binding
12505	3.3221E-2	63	207	endomembrane system
6164	3.3881E-2	29	79	purine nucleotide biosynthesis
6732	3.5041E-2	45	138	coenzyme metabolism
40008	3.5974E-2	38	112	regulation of growth
16575	3.6758E-2	7	10	histone deacetylation
9966	3.6758E-2	54	173	regulation of signal transduction
129	3.6758E-2	18	42	DNA-directed RNA polymerase activity
7582	3.6758E-2	2367	10844	physiological process
6468	3.6758E-2	144	541	protein amino acid phosphorylation
6357	3.6758E-2	74	252	regulation of transcription from RNA polymerase II promoter
51246	3.7219E-2	49	154	regulation of protein metabolism
15077	3.7866E-2	50	158	monovalent inorganic cation transporter activity

502	3.8236E-2	17	39	proteasome complex (sensu Eukaryota)
16070	3.8236E-2	113	412	RNA metabolism
3713	3.8847E-2	46	143	transcription coactivator activity
9987	3.9150E-2	2445	11225	cellular process
19001	4.2426E-2	95	339	guanyl nucleotide binding
6394	4.8004E-2	92	328	RNA processing
42623	4.8376E-2	73	251	ATPase activity, coupled
3702	4.8376E-2	65	219	RNA polymerase II transcription factor activity
279	4.8376E-2	66	223	M phase
6473	4.8590E-2	8	13	protein amino acid acetylation
4842	4.9930E-2	102	370	ubiquitin-protein ligase activity
